# Photoelectromagnetic Effect Induced by Terahertz Laser Radiation in Topological Crystalline Insulators Pb_1−*x*_Sn*_x_*Te

**DOI:** 10.3390/nano11123207

**Published:** 2021-11-26

**Authors:** Alexandra V. Galeeva, Dmitry A. Belov, Aleksei S. Kazakov, Anton V. Ikonnikov, Alexey I. Artamkin, Ludmila I. Ryabova, Valentine V. Volobuev, Gunther Springholz, Sergey N. Danilov, Dmitry R. Khokhlov

**Affiliations:** 1 Faculty of Physics, M.V. Lomonosov Moscow State University, 119991 Moscow, Russia; galeeva@physics.msu.ru (A.V.G.); belov.da17@physics.msu.ru (D.A.B.); askazakov@physics.msu.ru (A.S.K.); antikon@physics.msu.ru (A.V.I.); artamkin@mig.phys.msu.ru (A.I.A.); mila@mig.phys.msu.ru (L.I.R.); 2 Institute of Semiconductor and Solid State Physics, Johannes Kepler University of Linz, 4040 Linz, Austria; volobuiev@magtop.ifpan.edu.pl (V.V.V.); Gunther.Springholz@jku.at (G.S.); 3 International Research Centre Mag Top, Institute of Physics, Polish Academy of Sciences, PL-02668 Warsaw, Poland; 4 Department of Metals and Semiconductor Physics, National Technical University “KhPI”, 61000 Kharkiv, Ukraine; 5 Faculty of Physics, University of Regensburg, 93053 Regensburg, Germany; Sergey.Danilov@physik.uni-regensburg.de; 6 Department of Solid State Physics, P.N. Lebedev Physical Institute, 119991 Moscow, Russia

**Keywords:** topological crystalline insulator, terahertz radiation, photoelectromagnetic effect

## Abstract

Topological crystalline insulators form a class of semiconductors for which surface electron states with the Dirac dispersion relation are formed on surfaces with a certain crystallographic orientation. Pb_1−*x*_Sn*_x_*Te alloys belong to the topological crystalline phase when the SnTe content *x* exceeds 0.35, while they are in the trivial phase at *x* < 0.35. For the surface crystallographic orientation (111), the appearance of topologically nontrivial surface states is expected. We studied the photoelectromagnetic (PEM) effect induced by laser terahertz radiation in Pb_1−*x*_Sn*_x_*Te films in the composition range *x* = (0.11–0.44), with the (111) surface crystallographic orientation. It was found that in the trivial phase, the amplitude of the PEM effect is determined by the power of the incident radiation, while in the topological phase, the amplitude is proportional to the flux of laser radiation quanta. A possible mechanism responsible for the effect observed presumes damping of the thermalization rate of photoexcited electrons in the topological phase and, consequently, prevailing of electron diffusion, compared with energy relaxation.

## 1. Introduction

The physics of topological insulators is one of the rapidly developing areas of modern solid-state physics. In these materials, the energy positions of the conduction and valence bands in the bulk are inverted because of the strong spin–orbit interaction. As a consequence, conductive electron states with the Dirac dispersion relation and, consequently, zero effective mass necessarily appear at the surface (in the 3D case) or at the edge (in the 2D case) of a sample. Moreover, the spin direction of electrons being in these surface or edge electron states is locked perpendicular to their momentum direction, thus preventing backscattering, at least in theory [1,2,3]. Therefore, conductivity along the “topological” surface or edge channel is expected to be extremely high, which may be used in electronics.

In addition to topological insulators for which the existence of surface states is due to the time-reversal symmetry, there is another class of topologically nontrivial materials—topological crystalline insulators—for which the appearance of topological surface states is due to the symmetry of the crystal lattice [4,5,6,7]. In 3D topological insulators, topological surface states arise at all surfaces of the material, while in topological crystalline insulators, they form only at surfaces with a certain crystallographic orientation.

A classic example of a topological crystalline insulator is Pb_1−*x*_Sn*_x_*Te solid solutions [8]. These semiconductors are also widely known as good thermoelectric materials [9], emitters [10], and detectors [11,12] of infrared and terahertz radiation. In the composition region *x* < 0.35 of Pb_1−*x*_Sn*_x_*Te, the electronic energy spectrum is direct, the trivial phase is realized, and for *x* > 0.35, the spectrum becomes inverse, and the alloy moves into the phase of topological crystalline insulator [13,14].

In most cases, topologically non-trivial materials possess a high free carrier concentration in the bulk. Therefore, deriving the contribution of topological surface states to electron transport is a sophisticated task.

Another experimental approach is optoelectronic probing of topological electron states, which may be insensitive to the bulk conductivity. Examples of such phenomena include the photogalvanic effect [15,16,17,18], the photon-drag effect [19], and photoconductivity [20,21,22]. One more example of this kind is the photoelectromagnetic (PEM) effect.

The PEM effect appears in semiconductors upon photoexcitation in a magnetic field. For the “classical” PEM effect, the incident radiation excites electrons and holes in the surface layer of a semiconductor. The photoexcited free carriers diffuse into the semiconductor bulk. If magnetic field is applied parallel to the sample surface, i.e., the Voigt experimental geometry is realized, then the diffusing photoexcited carriers of the opposite sign are deflected by the Lorentz force to the opposite sample sides providing the appearance of a voltage drop, which is the PEM effect signal [23].

If the radiation quantum is smaller than the semiconductor bandgap, then the photoexcitation of electron–hole pairs may not be realized, and the incident radiation may only heat up the free carriers available. Then, the heated carriers start to diffuse into the material bulk, and the cold carriers in the bulk diffuse into the opposite direction to preserve electroneutrality. The appearance of the PEM effect signal in this situation depends on the difference in mobilities of the heated carriers at the surface and cold carriers in the bulk [24].

Previously, it has been demonstrated that high-mobility surface electron states in topological insulators [25], topological crystalline insulators [24], and Dirac semimetals [26] reveal themselves in the appearance of the PEM effect at low temperatures upon terahertz photoexcitation with the radiation quantum much less than the energy gap.

In this paper, this approach was applied to Pb_1−*x*_Sn*_x_*Te epitaxial films grown on a BaF_2_ substrate with the (111) surface crystallographic orientation, for which topological surface electron states are expected to arise [27,28,29,30,31].

## 2. Materials and Methods

Pb_1−*x*_Sn*_x_*Te epitaxial films were grown by molecular beam epitaxy on BaF_2_ substrates with the crystallographic orientation (111). The tin content in the films varied from *x* = 0.11 (direct spectrum, trivial phase) to *x* = 0.44 (inverse spectrum, topological phase), the thickness of all films was 1 μm. Samples with high SnTe content were additionally doped with Bi to decrease the free hole concentration that would normally arise in undoped samples due to increased deviation from stoichiometry in alloys with high *x* values [28]. Bismuth is known to be a strong donor that does not provide the appearance of impurity levels in the vicinity of the actual bands in Pb_1−*x*_Sn*_x_*Te and does not affect noticeably the band structure of the semiconductor alloy [13]. More details about sample growth and structural characterization can be found elsewhere [28,29,30].

For all samples, galvanomagnetic properties were characterized at temperatures *T* = (4.2–300) K through the resistivity and Hall effect measurements. The free carrier concentration *n*, *p* was on the order of 10^18^ cm^−3^, and all samples except for the one with *x* = 0.345 had the *p*-type conductivity. The mobility *μ* of charge carriers at *T* = 4.2 K was on the order of 10^4^ cm^2^/V⸱s, which is a quite high value for *p*-type Pb_1−*x*_Sn*_x_*Te alloys with high tin content. The main characteristics of the samples are presented in Table 1. The SnTe content *x* was determined by the XRD. The energy gap *E_g_* was calculated using the relation between *E_g_* and *x* [13,14]; the positive *E_g_* values correspond to the direct energy spectrum, and the negative values correspond to the inverse spectrum, for which the appearance of topological surface electron states is expected. The Fermi energy *E_F_* was calculated from the free carrier concentration value using the 2-zone Kane dispersion relation [14]. The *E_F_* values correspond to the energy distances between the Fermi level and the conduction band edge, for the *n*-type sample, and between the valence band edge and the Fermi level, for the *p*-type samples. For all samples, the Fermi level lies on the background of either valence (*p*-type) or conduction (*n*-type) band at low temperatures, i.e., the samples are degenerate semiconductors.

The photoelectromagnetic effect was measured using a pulsed terahertz NH3 gas laser optically pumped by transversely excited atmospheric pressure (TEA) CO2 laser. The pulse duration was about 100 ns, the power in a pulse was up to 5 kW, and the radiation wavelength was 90 μm or 280 μm.

The wavelength of the incident terahertz radiation corresponds to the Reststrahlen band of Pb_1−*x*_Sn*_x_*Te, i.e., the respective frequencies lie between the frequencies of the TO and LO phonons. In this frequency range, the radiation absorption is very high and is almost completely defined by the crystalline lattice. The estimates of the absorption length using the dielectric function of the coupled plasmon–LO phonon mode [32] yield values from several dozens to three-to-four hundred of nanometers, depending on the laser wavelength and the SnTe content *x*. This is mainly due to very high values of the static dielectric constant *ε* that well exceeds 10^4^, especially in alloys with the *x* value close to the band inversion point *x* = 0.35 [14]. Therefore, the radiation penetration length is much smaller than the film thickness.

**Figure 1:**
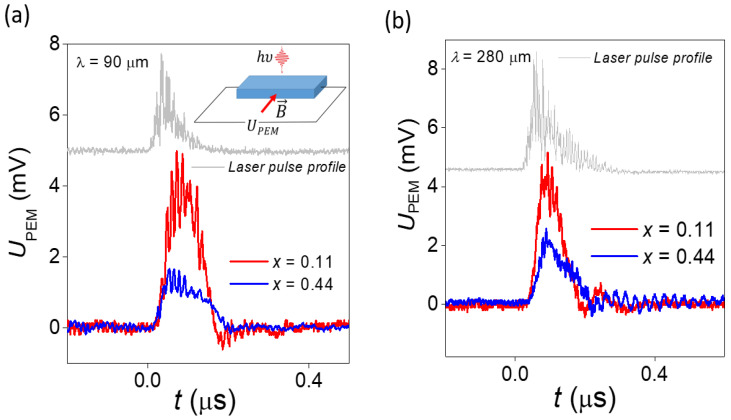
A typical PEM effect voltage kinetics measured in trivial (*x* = 0.11) and topological (*x* = 0.44) phase samples. Photoexcitation wavelengths *λ* = 90 μm (**a**) and 280 μm (**b**).

The radiation pulse kinetics and intensity were controlled by a photon-drag detector [33]. The radiation power could be varied by using calibrated attenuators. The sample was placed in an optical helium cryostat and was kept at the liquid helium temperature. The sample was centered in a superconducting solenoid, and measurements were carried out in magnetic fields up to 4 T. The Voigt experimental geometry was used. The experimental setup layout is shown in the inset to Figure 1a. More experimental details may be found in [34,35,36].

## 3. Results

The PEM effect signal was recorded for all samples. In all cases, the kinetics of the voltage pulse of the PEM effect repeated the kinetics of the laser pulse (Figure 1).

**Figure 2:**
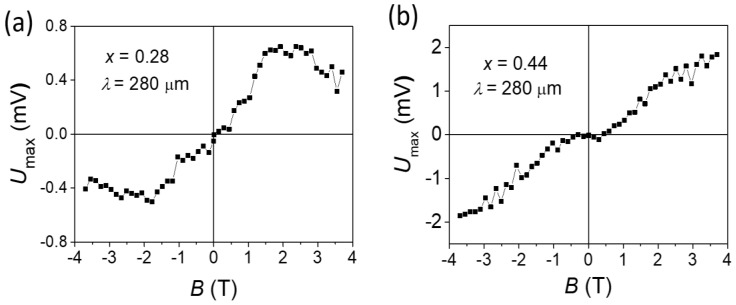
Typical magnetic field dependencies of the PEM effect amplitude in Pb_1−*x*_Sn*_x_*Te. Sample composition x = 0.28 (**a**) and 0.44 (**b**) corresponds to trivial and topological phases, respectively. Photoexcitation wavelength *λ* = 280 μm.

The effect was odd in the magnetic field, i.e., changed sign when the direction of the magnetic field changed, and in the zero field, it turned to zero. The amplitude of the effect increased with an increase in the magnetic field; for some samples, it reached a maximum and then slightly decreased (Figure 2). The magnetic field at which the maximum effect was observed varied from 1 to 3 T.

**Figure 3:**
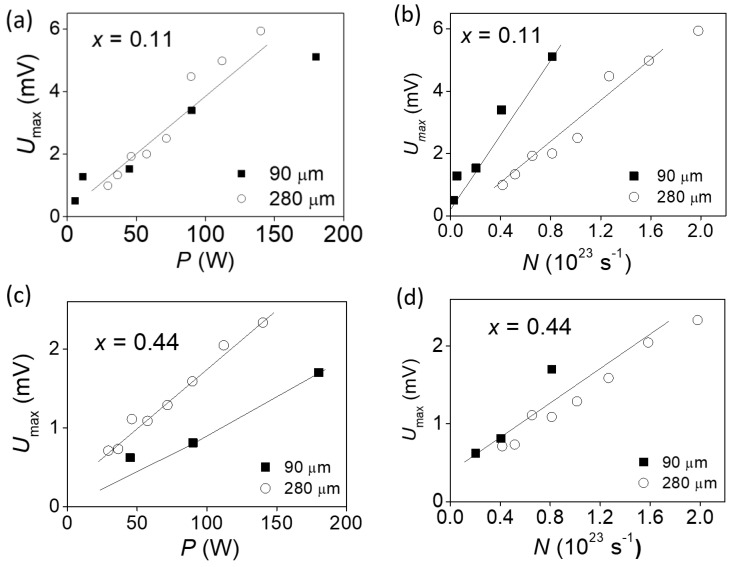
Dependence of the PEM effect amplitude on the radiation power (**a**,**c**) and incident quanta flux (**b**,**d**) for the trivial phase sample with *x* = 0.11 (**a**,**b**) and for the topological phase sample with *x* = 0.44 (**c**,**d**). Open symbols—radiation wavelength 280 μm, full symbols—90 μm. *B* = 0.44 T (**a**,**b**) and 3.7 T (**c**,**d**), *T* = 4.2 K.

Dependences of the PEM effect amplitude on the intensity of the incident radiation were measured. The measurements were carried out in a magnetic field in which the maximum amplitude of the effect is observed for each sample. It should be stressed that the measurements were performed for two very different laser wavelengths. It was found that for samples with a direct spectrum in the trivial phase, the PEM effect amplitude is defined by the power of radiation incident on a sample (Figure 3a,b). For the sample in the topological phase, the amplitude of the effect is proportional to the flux of radiation quanta incident on the sample (Figure 3c,d).

## 4. Discussion

**Figure 4:**
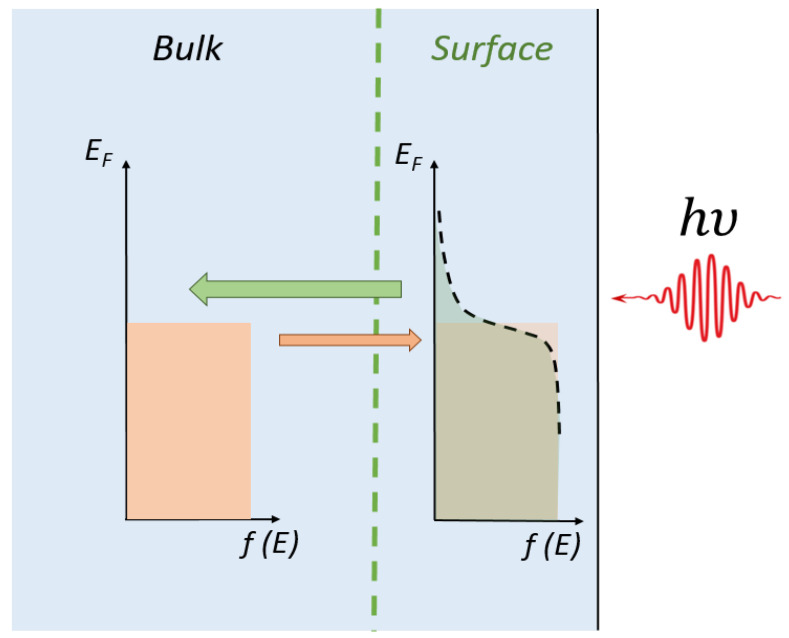
Schematic illustration of the mechanism leading to appearance of PEM effect in Pb_1−*x*_Sn*_x_*Te upon terahertz photoexcitation.

The mechanism of appearance of the PEM effect upon terahertz excitation is the following. Since the terahertz quantum energy is much less than the characteristic energies in the material spectrum, such as the energy gap, the terahertz excitation may not provide any extra free carriers in the semiconductor, it may only heat up already existing carriers in a surface layer of a sample. These carriers start to thermalize and diffuse to the sample bulk. At the same time, cold carriers diffuse from the sample bulk to the surface. If the diffusion rates of the two carrier fluxes are the same, the net diffusion current is zero, so the PEM effect may not appear. The presence of the PEM effect signal means that the diffusive flux from the surface to the bulk is higher than the carrier flux in the opposite direction (Figure 4). For degenerate semiconductors, the free carrier scattering time does not depend on energy at low temperatures, so the two fluxes must be compensated. The existence of the PEM effect signal may be due only to the appearance of free carriers with higher mobility on the sample surface.

Since the PEM effect is present for both trivial and topological phases of Pb1−xSnxTe, it is possible to conclude that the high-mobility electronic states are formed on the semiconductor surface in both cases. On the other hand, the effect intensity scales up in a different way for the trivial and topological phases—in the first case, it is proportional to the radiation power, while in the second case, it is proportional to the radiation quanta flux.

Such a difference may be due to different rates of thermalization of photoexcited charge carriers in the topological and trivial phases. In what follows, this process is considered in more detail.

The PEM effect signal formation may be considered as consisting of several steps. The first step is the excitation of electrons at the surface with monochromatic laser radiation. As a result, part of the surface electrons increases its energy by a certain amount, which equals the radiation quantum energy. The number of these electrons depends on the incident photon flux.

The next step is twofold, i.e., the excited electrons start to thermalize and diffuse to the sample bulk. The process scaling depends on the relation between the characteristic thermalization and diffusion times.

If the thermalization occurs faster, then the excited carriers first thermalize, thus obtaining a characteristic temperature, and only then start to diffuse. The characteristic temperature of photoexcited electrons is defined by the radiation power absorbed, so the effect scales up as a function of the incident power. Such a situation is apparently realized in the trivial phase.

If the thermalization is damped for some reason, then the diffusion process becomes faster, so the signal begins to scale up as a function of the incident photon flux. This situation corresponds to the topological phase.

The question that arises is why is the thermalization damped in the topological phase? A possible answer is the following: The thermalization rate is defined by the electron–electron interaction. Hot charge carriers in the topological phase are thermalized much slowly, which is due to the rigid binding of the direction of the electron spin to the direction of its momentum [23,24]. Therefore, the number of effectively interacting electrons sharply decreases in comparison with the trivial phase and is determined only by the number of electrons with the same direction of the momentum and, accordingly, the electron spin.

In the trivial phase, the photoexcited electron relaxation is much faster due to the higher number of effectively interacting electrons. As a result, the electrons first thermalize in the surface layer and only then start to diffuse to the sample bulk.

Two important points should be noted here. First, the characteristic times of all these processes are on the order of hundreds of femtoseconds to picoseconds, which is much shorter than the characteristic laser pulse length of about 100 ns. Therefore, thermalization and diffusion are not observable in the effect kinetics. Second, the radiation penetration depth does not need to be on the order of the surface layer thickness for the PEM effect appearance mechanism described above. It is only necessary that some noticeable part of the incident radiation is absorbed in the surface high-mobility electron layer. This is true even for monolayer graphene, for which 2.3% of the incident radiation is absorbed [37]. The topological layer is expected to be at least 10 times thicker, so one could expect about 20% of the radiation absorbed in it.

It should be emphasized that the damping effect of hot electron thermalization appears as a specific signature of topologically non-trivial surface states. It is observed in topological insulators [25], Dirac semimetals [26], and topological crystalline insulators (present study), at least for the (111) crystallographic surface orientation, at which topological surface electron states are expected to be observed.

## 5. Conclusions

In conclusion, it has been demonstrated that the PEM effect amplitude scales up as the incident terahertz radiation power for the trivial phase of Pb_1−*x*_Sn*_x_*Te films, whereas it is proportional to the incident photon flux for the topological phase samples. This result is explained in terms of damping of the thermalization process of free carriers heated by terahertz pulses, in the topological phase. Thus, the obtained results are an argument in favor of the fact that a topological state is in fact realized on the surface (111) in the topological phase Pb_1−*x*_Sn*_x_*Te. The effect is a common feature of semiconductor systems possessing non-trivial topological surface electron states.

## Figures and Tables

**Table 1:** Characterization of the Pb_1−*x*_Sn*_x_*Te samples studied. The SnTe content *x* was determined by the XRD; the energy gap *E_g_* was calculated using the *x* value as described in the text; the Bi content was determined by controlling the Bi flux in the MBE setup; the carrier type, resistivity *ρ*, free carrier concentration *n*, *p*, carrier mobility *μ* were determined through the resistivity and Hall effect measurements; position of the Fermi energy *E_F_* with respect to the band edges was calculated using the Kane dispersion relation as described in the text.

*x*, mol.%	*E_g_*, meV	Carrier Type	Bi Content, at.%	*ρ*, 10^−4^ Ohm⸱cm, *T* = 4.2 K	*n*, *p*, 10^18^ cm^−3^, *T* = 4.2 K	*μ*, cm^2^/V⸱s, *T* = 4.2 K	*E_F_*, meV
0.11	130	*p*	0	3.7	1.1	15,300	33
0.28	40	*p*	0	1.8	2.1	16,400	79
0.345	2	*n*	0.02	5.1	1.0	12,200	70
0.44	−51	*p*	0.76	1.7	4.6	8200	70

## Data Availability

The data are available from the corresponding author upon a reasonable request.
